# Practical Analysis of IEEE 802.11n 2.4 GHz Communication Quality in the Context of IoT Devices Operating in an Area Shared with Modern Wi-Fi 6 and Wi-Fi 7 Networks

**DOI:** 10.3390/s26175352

**Published:** 2026-08-24

**Authors:** Andrzej Zankiewicz

**Affiliations:** Faculty of Electrical Engineering, Bialystok University of Technology, Wiejska 45D, 15-351 Bialystok, Poland; a.zankiewicz@pb.edu.pl

**Keywords:** end-to-end delay, goodput, IEEE 802.11n, Internet of Things (IoT), jitter, MQTT, packet delivery ratio (PDR), Wi-Fi 6 (IEEE 802.11ax), Wi-Fi 7 (IEEE 802.11be), Wi-Fi coexistence

## Abstract

This article presents an experimental evaluation of the data-transmission performance of an IoT device communicating with an MQTT broker over a local IEEE 802.11n Wi-Fi network in the 2.4 GHz band, coexisting with modern Wi-Fi networks based on the IEEE 802.11ax (Wi-Fi 6) and IEEE 802.11be (Wi-Fi 7) standards. The effect of sharing the radio medium on the communication quality parameters of the IoT device, such as end-to-end delay, jitter, the packet delivery ratio (PDR), and application throughput (goodput), was assessed. Unlike previous work, which focuses primarily on PHY/MAC metrics or on simulation-based analysis, this study targets the application layer (MQTT) from the perspective of a legacy IoT end-device. The results presented and discussed herein show that the presence of 802.11ax and 802.11be networks in the 2.4 GHz band can significantly degrade the temporal parameters of MQTT transmissions performed by IoT devices operating in the older 802.11n standard. The greatest impact is observed in jitter and in the extreme delay values (for instance, in the worst-case coexistence scenario—with simultaneous Wi-Fi 6 and Wi-Fi 7 interference, the P95 delay increased from 38 to 869 ms), whereas the PDR remains relatively high because retransmissions compensate for packet losses at the expense of delay.

## 1. Introduction

The Internet of Things (IoT) has become one of the key areas of contemporary information and communication systems, encompassing both simple sensor networks and complex industrial environments, smart buildings, monitoring systems, and applications within urban infrastructure. In many such use cases, what matters is not only the mere capability of wireless data exchange but also the predictability of latency, the stability of message delivery times, and the robustness of communication against interference and shared use of the medium. This is particularly relevant in monitoring, control, and near-real-time event analysis applications [1].

The rapid growth of the Internet of Things means that an increasing number of end-devices communicate in wireless environments characterized by high traffic density, sharing the same radio band with other transmission systems. In practice, a significant proportion of IoT devices communicate over local Wi-Fi networks, particularly in the 2.4 GHz band. This results from the low cost of radio modules, the wide availability of infrastructure, good indoor propagation, and the backward compatibility of successive generations of the IEEE 802.11 standard [2].

At the same time, this band is heavily loaded, as it is shared by devices operating in different generations of the IEEE 802.11 standard, as well as by other radio technologies [3,4]. The IEEE 802.11n standard, in particular, retains a remarkably persistent role in embedded solutions and telemetry devices. It introduced, among other things, MIMO techniques, frame aggregation, and mechanisms improving transmission throughput and range, while remaining compatible with earlier generations of Wi-Fi devices [5]. For many economical and long-lived IoT devices, this standard still constitutes a practical basis for radio access.

At the same time, the radio environment in which contemporary IoT devices operate is currently undergoing a significant transformation. Successive generations of wireless networks based on IEEE 802.11ax (Wi-Fi 6) [6] and IEEE 802.11be (Wi-Fi 7) [7] have been designed not only with the aim of increasing throughput but above all to improve spectral efficiency, reduce latency, and provide better support for dense and heterogeneous environments. This means that older devices operating in the 802.11n standard increasingly share the radio medium with infrastructure and terminals employing newer transmission mechanisms, which have been optimized for different traffic profiles, different load conditions, and a larger number of simultaneously active stations.

The IEEE 802.11ax standard [6] was developed primarily with a view to improving the operation of WLANs in environments with a high density of users and overlapping radio cells. As the survey [8] shows, the key mechanisms of 802.11ax include OFDMA, MU-MIMO, spatial reuse mechanisms, BSS coloring, and Target Wake Time, which together are intended to increase spectral efficiency, reduce medium-access overhead, and improve airtime utilization. Consequently, Wi-Fi 6 should be regarded not merely as an evolution increasing theoretical throughput but as a set of solutions that organize and optimize the operation of dense, coexisting radio environments [9]. The next stage in the development of Wi-Fi is defined by the IEEE 802.11be (Wi-Fi 7) standard [7], whose design objectives place even stronger emphasis on the need for very high throughput, low latency, greater reliability, and more flexible use of the spectrum. Wi-Fi Alliance documents, vendors’ technical guides, and recent review works indicate that the most important Wi-Fi 7 mechanisms include, among others, Multi-Link Operation (MLO), 4K-QAM, wider channels, multiple resource units, enhanced block acknowledgements, and solutions improving the handling of time-sensitive applications [10,11,12].

In deployment practice, however, it is essential to note that the new 802.11ax mechanisms must operate in mixed environments in which legacy stations, including IEEE 802.11n devices, are still present. Studies on the coexistence of 802.11ax networks with legacy devices indicate that mixed environments constitute one of the most important practical and research challenges of successive WLAN generations. In [13], the authors showed that in mixed 802.11ax networks involving older stations the overall performance of the radio environment deteriorates, and the beneficial impact of mechanisms such as BSS coloring may be limited by backward compatibility and by the behavior of terminals that do not support the new features. The authors also demonstrated that classical metrics such as throughput, average delay, or packet loss in mixed environments may differ significantly from the results obtained in homogeneous scenarios. This means that mixed 802.11ax/legacy environments should be treated as a distinct and not yet fully resolved research problem, especially given that the transition period between technology generations will entail a prolonged coexistence of modern infrastructure with earlier clients and end-devices [14].

From the perspective of IoT systems, the features of the new Wi-Fi standards have a two-fold significance. On the one hand, modern 802.11ax and 802.11be networks are designed to increase spectral efficiency, reduce collisions, and better manage radio resources in multi-station environments. On the other hand, in real-world deployments IoT devices very often do not directly benefit from the new capabilities of these standards, because they are still equipped with simpler 802.11n interfaces or compatible 2.4 GHz chipsets with limited capabilities. In practice, this leads to a situation in which telemetry devices with modest hardware requirements share the radio medium, with modern networks generating more complex and dynamic traffic [12,14].

In addition to wireless transmission, IoT systems use various application-layer protocols. One of the most widely used communication protocols in IoT systems is MQTT (Message Queuing Telemetry Transport). According to the OASIS specification [15], it is a lightweight publish/subscribe protocol intended to operate in resource-constrained environments, running over an ordered and reliable transport connection, most commonly TCP/IP, and offering three levels of quality of service (QoS) for data delivery. The survey [16] indicates that MQTT has become one of the dominant protocols for M2M and IoT communication owing to its implementation simplicity, low transmission overhead, and broad ecosystem support on the part of brokers and client libraries.

This article presents a practical analysis of the set of elements mentioned above: the effectiveness of data transmission of an IoT device communicating with an MQTT broker (Eclipse Mosquitto 2.1.2) over a local IEEE 802.11n Wi-Fi network in the 2.4 GHz band while coexisting with modern IEEE 802.11ax and IEEE 802.11be networks. In contrast to many earlier works focused on network metrics or on the potential of the new standards, this study is oriented toward the quality of application-layer communication from the perspective of the IoT device and the MQTT broker. End-to-end delay, jitter, the packet delivery ratio (PDR), and application throughput were evaluated, which makes it possible to capture both the reliability and the temporal stability of the transmission. Such an approach enables a better understanding of the practical consequences of Wi-Fi network modernization for existing IoT devices that use older radio interfaces.

This paper is organized as follows. Section 2 reviews relevant research and identifies the research problem. Section 3 describes the experimental methodology: Section 3.1 presents the structure of the test environment and hardware used; Section 3.2 defines the quality metrics used in the experiments and their measurement procedure; Section 3.3 specifies four experimental scenarios and their radio channel configurations; and Section 3.4 discusses the measurement of one-way delay and an approach to time synchronization between the transmitter and the receiver. Section 4 presents the experimental results, including empirical functions of the cumulative MQTT delay distribution, jitter and goodput characteristics, and packet deliverability for all analyzed scenarios. Section 5 discusses the observed coexistence effects in the context of the IEEE 802.11 family of standards, with a particular focus on media access asymmetry in mixed-standard environments and practical implications for IoT system design, standards evolution, and audits and acceptance tests of IoT systems. Chapter 6 summarizes the main scientific conclusions, identifies the limitations of the study, and identifies directions for future work.

## 2. Related Work

The existing literature indicates that the analysis of IoT communication quality cannot be confined either to application-layer metrics (e.g., MQTT) or to PHY/MAC parameters alone. Studies devoted to the evaluation of MQTT systems show that the resulting communication quality depends not only on the QoS level and the broker’s operating logic but also on the characteristics of the access layer, on network delays, packet losses, and jitter, and on the location of the data-processing elements. In particular, it has been shown that changes in network conditions can significantly affect end-to-end delay, response time, and transmission stability, and hence the actual usability of the system in time-sensitive applications [17,18,19,20].

In turn, studies concerning 802.11ax and mixed environments indicate that the presence of legacy devices can lead to reduced throughput, increased delays, and disruption of the mechanisms designed to improve network efficiency. This means that in MQTT-based IoT systems, parameters such as end-to-end delay, jitter, the packet delivery ratio (PDR), and goodput should be analyzed jointly, since maintaining a high PDR may occur at the expense of retransmissions and increased delay and therefore need not imply that good temporal transmission quality is preserved [21,22].

This phenomenon has been confirmed both in experimental studies of MQTT architectures in edge/cloud environments and in works comparing MQTT with other protocols used in the IoT, such as CoAP [23] or OPC UA [24]. The results of these analyses indicate that MQTT offers a favorable trade-off between functionality, ecosystem maturity, and communication overhead, while at the same time its temporal parameters are sensitive to the degradation of network conditions and to the configuration of the transport and application layers. In other words, a high effectiveness of logical message delivery does not automatically entail low latency or low variability in delivery times [25,26,27,28,29,30,31].

In the context of wireless communication for the IoT, quality metrics such as end-to-end delay, jitter, the packet delivery ratio (PDR), and application throughput (goodput) therefore play a particularly important role. While in many environmental monitoring applications the loss of a single sample may be acceptable, increased delay variability and irregular message delivery times can substantially degrade the operation of control systems, event-detection mechanisms, process synchronization, or data fusion algorithms. At the same time, retransmissions at the link and transport layers can keep the PDR at a relatively high level but at the cost of longer delivery times and increased delay dispersion. This general relationship is confirmed by works devoted to testing MQTT brokers and the performance of IoT protocols, in which a clear influence of network delay, jitter, and packet loss on the final system performance has been demonstrated [32,33].

The literature review presented above demonstrates that, despite the growing number of studies, a clear research gap exists at the intersection of three areas: (i) real-world experiments with MQTT communication of IoT devices, (ii) operation in the 2.4 GHz band using the older IEEE 802.11n standard, and (iii) coexistence with modern IEEE 802.11ax and, in particular, IEEE 802.11be networks. Available studies on mixed 802.11ax/legacy networks focus mainly on simulation-based analysis or on MAC/PHY-layer metrics, often in the 5 GHz band, whereas the literature devoted to the MQTT most frequently examines the influence of system architecture, transport type, broker location, or QoS level, rather than directly addressing the effects of sharing the radio medium with modern Wi-Fi generations. In the case of Wi-Fi 7, a considerable part of the publications is still of a review, conceptual, or preliminary assessment nature, focusing on potential benefits such as MLO and latency reduction rather than on the impact of the new networks on older IoT devices operating in the same band.

In summary, the following three gaps in the existing literature were identified, which together define the specific gap that this study fills:

- Studies on mixed 802.11ax/legacy environments (e.g., [13,14]) focus primarily on simulation-based analysis or on MAC/PHY-layer throughput metrics, typically in the 5 GHz band, without evaluating the effect on the application-layer MQTT communication of IoT devices.
- Studies devoted to MQTT performance (e.g., [17,18,19,20]) examine the influence of system architecture, broker location, transport type, or QoS level but do not address the direct impact of sharing the 2.4 GHz medium with HE-generation networks.
- For Wi-Fi 7 (IEEE 802.11be), the available publications remain predominantly of a review, conceptual, or preliminary assessment nature; to the best of the author’s knowledge, no direct experimental measurement of the impact of 802.11be networks on the MQTT communication quality of legacy 802.11n IoT devices has been reported in the literature.

For this reason, it is justified to undertake experimental research that will make it possible to assess to what extent the presence of modern WLANs affects the actual quality of MQTT transmission carried out by an IoT device operating in the IEEE 802.11n standard in the 2.4 GHz band. Such a problem is of both cognitive and practical significance, since many existing deployments will continue to operate for years in an environment of gradual infrastructure migration to Wi-Fi 6 and Wi-Fi 7. It therefore becomes crucial to determine whether, in coexistence scenarios, the degradation of quality manifests itself primarily as a decrease in the PDR, as a reduction in goodput, or rather as an increase in delay and jitter, which may be less apparent in classical reliability metrics yet more detrimental to near-real-time applications. The research problem formulated in this way is directly aligned with the current state of development of wireless networks and with the practical challenges of designing IoT systems in heterogeneous environments.

## 3. Materials and Methods

### 3.1. Structure of the Test Environment

**Figure 1:**
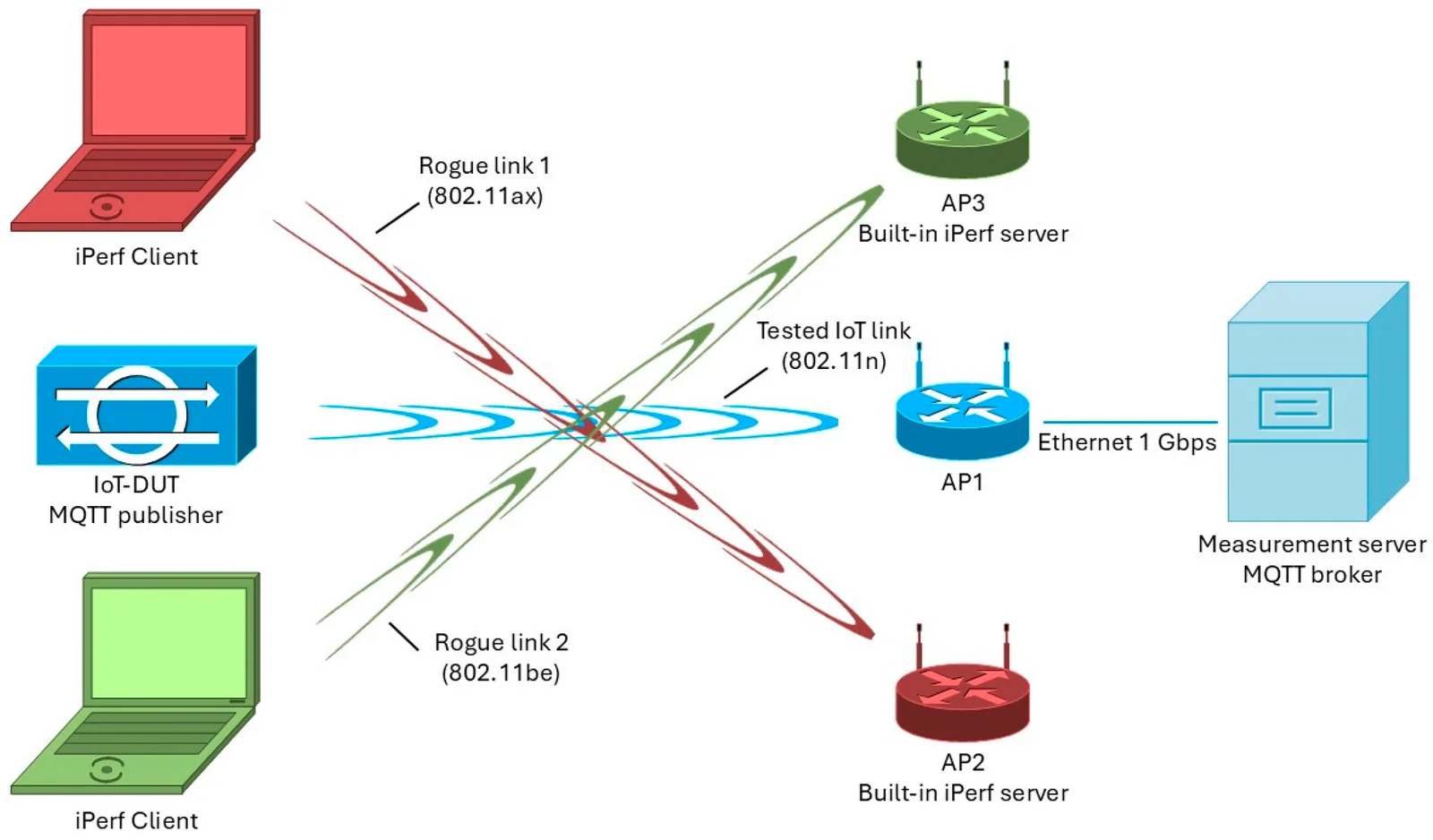
Block diagram of the test environment.

The experimental studies were carried out in a test environment whose structure is presented in Figure 1.

This environment comprises three Wi-Fi networks operating in the same room with an area of approximately 70 m^2^. For the duration of the experiments, all Wi-Fi networks not involved in the experiments were turned off in this room and in all the adjacent rooms, and the lack of interference was verified using the Wi-Fi analyzer mobile application. The first Wi-Fi network provides the radio link between the tested IoT device (hereinafter denoted IoT_DUT—Device Under Test) and its dedicated access point (AP1). This link operates in the IEEE 802.11n standard on channel 6 in the 2.4 GHz band. The channel width was set to 20 MHz, which corresponds to the actual operating parameters of typical IoT devices. The two remaining Wi-Fi networks (Rogue link 1 and Rogue link 2) are networks that interfere with the communication of the IoT_DUT device. They operate in the 802.11ax (Wi-Fi 6) and 802.11be (Wi-Fi 7) standards, respectively, with a channel width of 20 MHz, and are formed by AP2 (MikroTik hAP ax^3^, SIA Mikrotīkls, Riga, Latvia) and AP3 (Ubiquiti U7 Pro Wall, Ubiquiti, New York, NY, USA) together with PC stations generating traffic that fully loads the interfering Wi-Fi link. This traffic was generated using iperf3 software installed on the PC stations (iPerf client) and built into the access-point systems (iPerf server) [34].

An ESP32 DevkitC-32E module (Espressif Systems, CO., Ltd, Shanghai, China) was used as the IoT_DUT, and a MikroTik hAP ac^2^ device (SIA Mikrotīkls, Riga, Latvia) was employed as its dedicated access point (AP1). ESP32 DevkitC-32E is one of the most widely deployed microcontroller platforms in IoT development globally, with an integrated 802.11b/g/n radio operating in the 2.4 GHz band. The 802.11n STA mode with a 20 MHz channel width is representative of the actual operating parameters of low-cost IoT devices currently in production. MikroTik hAP ac^2^ supports IEEE 802.11n in the 2.4 GHz band in AP mode with a configurable channel and width and without proprietary traffic shaping that could distort the measured delays. Both devices are commercially available and reproducible hardware and represent appropriate device categories for the assumed research concept.

This AP1 access point is connected via a 1 Gbps Ethernet LAN to a server running the MQTT broker and implemented on Mosquitto software [35]. Owing to the placement of the MQTT broker within the local Ethernet network, the influence of the delays of any potential WAN (e.g., the Internet) was minimized, so that the measured delays primarily reflect the impact of the radio and MAC layers. Table 1 summarizes the complete experimental setup configuration.

The IoT_DUT device has software implemented that configures the device’s Wi-Fi interface in the 802.11n STA mode, connects to the Wi-Fi network advertised by AP1, and cyclically publishes to the MQTT broker messages containing a sequence number (incremented by one for each transmitted message) and a timestamp (Unix epoch time). These messages are published cyclically to the MQTT topic *test/up* with a period configured as a parameter in the IoT_DUT device software.

### 3.2. Research Methodology

The studies were carried out in the test environment, whose topology is described in Section 3.1. During the experiments, a subscription to the *test/up* topic from the MQTT broker was performed by software written in Python (version 3.13.7) and running on the measurement server. Each message sent by the IoT_DUT to the MQTT broker contained a sequence number *seq* and a transmission timestamp $t_{tx}^{iot}$ obtained from the device clock, set immediately before invoking the function that publishes the MQTT message. On the measurement server side (the MQTT subscriber), at the moment a message was received, the time $t_{rx}^{sub}$ was recorded, and the following were computed:

(i) The one-way delay (OWD) $d_{i}\;\;$ at the level of the application running on the measurement server:

(1) $$d_{i}=t_{rx,\;i}^{sub}-\;t_{tx,\;i}^{iot}\;$$

(ii) The application-layer jitter:

(2) $$J_{i}=\left|d_{i}-\;d_{i-1}\right|$$

The time $t_{tx,\;i}^{iot}$ was stamped on the IoT_DUT using the *gettimeofday()* function returning Unix epoch time in ms, invoked immediately before publishing the message. The time was stamped in the application-layer MQTT subscriber at the moment the message was delivered to the subscriber’s callback function.

Based on the messages received from the MQTT broker, the software running on the measurement server generated a CSV file containing records in the following format:

scenario,id,seq,t_tx_ms,t_rx_ms,raw_ms,offset_ms,owd_ms,lost,goodput_kbps,drift_ms_per_s

The individual components of a row in the CSV file contain:

scenario—the name of the tested scenario (used, among others, in the charts);
id—the identifier of the IoT_DUT module (e.g., esp32_1);
seq—the sequential number of the message sent by the IoT_DUT (incremented with each transmission);
t_tx_ms—The timestamp of the message transmission (Unix epoch);
t_rx_ms—The timestamp of the message reception (Unix epoch);
raw_ms—The difference between the reception and transmission times of the message;
offset_ms—The correction of the differences between the receiver and transmitter clocks (determined during the calibration phase, described in Section 3.4);
owd_ms—The determined one-way delay;
lost—A true/false value indicating the absence of an expected message;
goodput_kbps—The average transmission rate determined over a 60 s window;
drift_ms_per_s—The change (drift) of the one-way delay (owd) determined over a 1 s window.

In the subsequent steps, the data stored in the CSV files were used to compute and plot the cumulative distribution functions of the delays, the jitter values, the application throughput, and the reliability indicators. Ultimately, the following metrics were defined:

- OWD delay: $d_{i}$ with the appropriate correction (Section 3.4);
- Application-layer jitter (Equation (2));
- Application goodput: the number of payload bits correctly delivered per unit of time;
- PDR (packet delivery ratio): *PDR* = *Nd/N*;

Where *Nd* is the number of correctly delivered messages and *N* is the number of all sent messages.

These four metrics collectively characterize the communication quality from both reliability and temporal perspectives, which is necessary for a complete assessment of IoT service quality:

- OWD (one-way delay): the primary metric for latency in IoT applications; defined and standardized in IETF RFC 7679 [36]; directly reflects the time between a sensor event and its receipt by the application.
- Jitter (inter-message delay variation): critical for applications requiring temporal regularity (control loops, data fusion, and time-synchronized sampling); a high PDR with high jitter indicates unreliable temporal behavior.
- PDR (packet delivery ratio): the classical reliability metric for MQTT/IoT systems [16,21,22]; captures message loss due to MAC/TCP retransmission limit exhaustion.
- Goodput: the application-level throughput, capturing both the PDR and delay effects on the effective information transfer rate; reveals throughput instability not visible in average metrics.

As shown in [21,22,32], these four metrics together can capture the scenario in which a high PDR masks temporal quality problems, a central finding of the present study.

It should be emphasized that $d_{i}$ is the one-way MQTT application-layer delay, encompassing: access to the Wi-Fi medium, transmission in the MAC/PHY layer, TCP/IP transport, processing in the MQTT broker, and handling in the subscriber client. Such a definition is appropriate for assessing the quality of IoT services, since it directly reflects the delay perceived by the end application. In MQTT, a message is carried over an ordered, lossless network stream (typically TCP), so the time of its arrival at the application depends on the delays, buffering, and retransmissions of the lower layers. The uncertainty in measuring the one-way delay arises mainly from (i) clock-synchronization deviations (offset and drift) and (ii) the timestamping delay in the software (OS Windows 11 25H2 queues, system scheduler).

**Figure 2:**
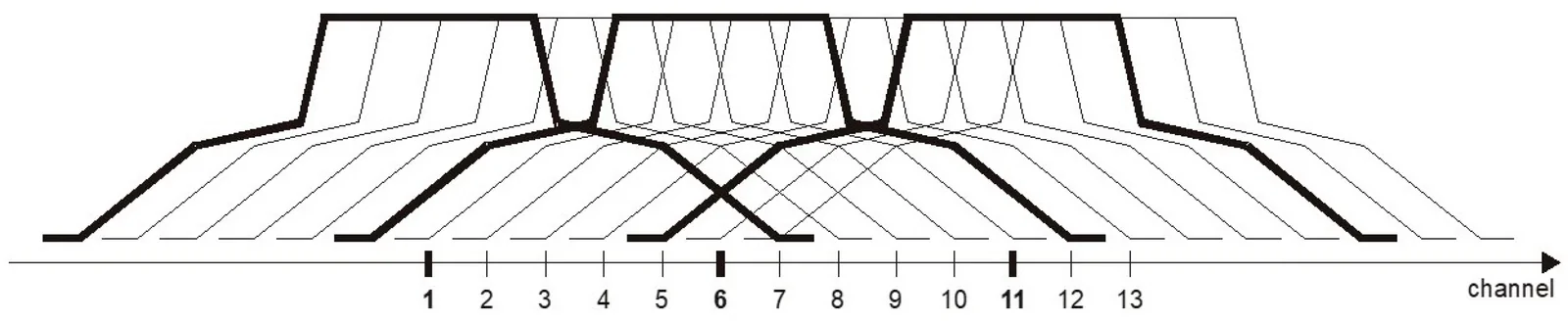
Spectral masks of the frequency channels in the 2.4 GHz band.

The frequency channels of the particular networks were selected so as to cover cases ranging from full overlap of the channel of the tested network and the interfering network to a complete absence of overlap between the main lobes of these networks. Figure 2 shows the spectral masks of the channels in the 2.4 GHz band.

It follows from this figure that, in order to satisfy the assumed requirement regarding channel overlap and assuming that the tested IoT_DUT network operates on channel 6, the interfering network should vary its channel in the range from 1 to 11. The choice of channel 6 for the IoT_DUT is justified by the following: (i) channel 6 is one of the three standard non-overlapping channels in the 2.4 GHz band (channels 1, 6, 11) most commonly used in real-world Wi-Fi deployments, and (ii) fixing the DUT on channel 6 provides a symmetric coverage of all possible overlap conditions, as when the interfering network’s channel varies from 1 to 11, the spectral overlap ranges from zero (channels 1 and 11) through partial (channels 2–5 and 7–10) to full (channel 6).

It is worth noting that modern Wi-Fi infrastructure supports several mechanisms for adaptive RF channel and band management, including Automatic Channel Selection (ACS), IEEE 802.11k/v-based band steering, DFS (in the 5 GHz band), IEEE 802.11ax BSS coloring, and Wi-Fi 7 Multi-Link Operation. In principle, these mechanisms could partially alleviate coexistence issues in mixed-standard environments. However, in the context of legacy IEEE 802.11n IoT devices of the class studied in this paper, their applicability is severely constrained. First, low-cost IoT microcontrollers are typically single-band 2.4 GHz devices and are physically incapable of band switching; band steering mechanisms are therefore ineffective for this device class. Second, 802.11k/v support is typically absent from lightweight IoT Wi-Fi stacks. Third, in practice, the channel of the IoT access point is often statically configured by the network administrator to ensure stable, predictable connectivity, and dynamic channel reassignment at the AP level would disrupt ongoing MQTT/TCP sessions. Consequently, the coexistence degradation effects documented in this paper represent conditions that are largely unavoidable for the installed base of legacy 802.11n IoT devices—adaptive channel management mechanisms provide relief primarily for modern, dual-band, full-featured client stations, not for the resource-constrained, single-band IoT end-devices that form the subject of this study. This observation further reinforces the practical significance of the findings: quantifying the impact of HE-network coexistence on legacy IoT devices is essential precisely because these devices cannot autonomously mitigate it.

### 3.3. Research Scenarios

Studies were conducted in the four scenarios described below, which also included various variants of configurations of the radio channels. For each scenario and its measurement variant, a series of 30 min experiments was performed, during which the IoT_DUT device published MQTT messages at a rate of 1 Hz with a payload size of 256 Bytes at QoS 0. In each tested variant of a given scenario, 1800 measurement transmission trials were performed. For each trial, the one-way delay (OWD), the occurrence of message loss, the application-layer jitter, and the effective application throughput (goodput) were recorded.

Scenario_1: only the IoT_DUT device is present, with no interfering Wi-Fi networks;

Scenario_2: the IoT_DUT device (channel 6) is present together with an interfering Wi-Fi 6 (IEEE 802.11ax) network operating on a channel varying from 1 to 11 (eleven measurement variants);

Scenario_3: the IoT_DUT device (channel 6) is present together with an interfering Wi-Fi 7 (IEEE 802.11be) network operating on a channel varying from 1 to 11 (eleven measurement variants);

Scenario_4: the IoT_DUT device (channel 6) is present together with two interfering networks (Wi-Fi 6 and Wi-Fi 7) operating, respectively, on the channel pairs (1, 11), (3, 9), (4, 9), (5, 9), (6, 9), (7, 9), (8, 9), (9, 9), (4, 8), (5, 8), (6, 8), (7, 8), (8, 8), (9, 8), (4, 7), (5, 7), (6, 7), (7, 7), (8, 7), (9, 7), (4, 6), (5, 6), (6, 6), (7, 6), (8, 6), and (9, 6)—twenty-six measurement variants.

In the case of Scenario_4, the channel pairs listed above, on which the full measurements were performed, were selected on the basis of short (a few minutes) rough measurements, so that the full results would cover a representative range of interference, spanning cases from the absence of overlap of the individual interfering networks up to their full overlap with the tested network. Table 2 summarizes the configuration of all experimental scenarios.

The four scenarios were designed to systematically cover the most practically relevant coexistence configurations encountered in modern indoor wireless environments:

- Scenario_1 (no interference): establishes the baseline communication quality of the 802.11n IoT device without coexistence effects; corresponds to a legacy Wi-Fi deployment or to an environment where IoT devices operate on a dedicated SSID/AP.
- Scenario_2 (802.11ax interference): represents the most common current deployment scenario: a single modern Wi-Fi 6 access point sharing the 2.4 GHz band with an existing IoT device; allows direct quantification of the isolated effect of Wi-Fi 6.
- Scenario_3 (802.11be interference): represents the increasingly common scenario as Wi-Fi 7 equipment enters the market; the isolated effect of Wi-Fi 7 can be directly compared with Wi-Fi 6 (Scenario_2).
- Scenario_4 (simultaneous 802.11ax + 802.11be interference): represents a dense heterogeneous environment (e.g., modern office, industrial building, residential block) where multiple HE networks co-exist; constitutes the worst-case practically relevant scenario.

Through the channel arrangement presented above, each of the tested measurement scenarios covered cases ranging from a complete overlap of the interfering network’s channel with the channel of the IoT_DUT device (channel pair (6, 6)) up to a minimal overlap of the channels of the tested and interfering networks.

### 3.4. The Issue of Time Synchronization Between the Transmitter and the Receiver

Since the primary measurement result is the one-way transmission delay from the transmitter (IoT_DUT) to the receiver (the measurement server), its precise determination requires reliable time synchronization between these two endpoints.

In the studies, the one-way delay $d_{i}$ (OWD) was measured at the level of the MQTT application, defined as the difference between the time a message is received in the application-layer subscriber (on the server side) and the time the message is sent by the IoT_DUT device (Equation (1)). To enable the correct determination of $d_{i}$, the clocks of the IoT_DUT device and of the measurement server were synchronized to a common time scale via the NTP protocol and a local time server. The sending and receiving of messages were performed only after the time synchronization procedures had been completed and, additionally, the time offset between the receiver and the transmitter had been determined. The clock offset between the IoT_DUT and the MQTT subscriber host (the measurement server) was estimated as the median of the differences $t_{rx}^{sub}-\;t_{tx}^{iot}\;$ over a synchronization window comprising *N* samples. This offset was then subtracted from subsequent samples, which eliminates the synchronization error without affecting the relative delay values or their statistical characteristics.

In the analysis of the results, it was taken into account that the OWD measurement error is the sum of the synchronization error and the timestamping error on both sides, that is

(3) $$OWD=\left(t_{rx,\;i}^{sub}-t_{tx,\;i}^{iot}\right)-\hat{\Delta }=d_{i}-\hat{\Delta }$$

where $\hat{\Delta }$ is the clock offset estimated over a window of *N* samples, computed as

(4) $$\hat{\Delta }=median(t_{rx}^{sub}-t_{tx}^{iot})$$

The use of the median rather than the mean is justified by the fact that the jitter does not have a distribution symmetric around zero, and the delay distribution has a long right tail (the trials showed that individual TCP and Wi-Fi MAC retransmissions can add 50…300 ms, and, in addition, the Windows scheduler and the TCP stack contribute their own momentary “spikes”), so that the mean value is not an unbiased estimator of the offset. The median is a solution frequently used in network delay measurements [36,37,38].

The NTP residual synchronization error between the ESP32 DevkitC-32E and the measurement server was estimated at 1–5 ms under the experimental conditions, based on multiple calibration rounds conducted before and after the measurement campaign. This uncertainty is negligible relative to the measured delay values (typically 10–2500 ms). The frequency of synchronization was studied as follows. NTP synchronization was performed once at the beginning of each 30 min experiment. For multi-hour measurement sessions, an optional background re-calibration could be applied via the automatic plateau-monitoring. Stability was verified by the plateau criterion: the calibration was considered complete when, for the chosen *N*, the median offset changed by less than 2 ms for 10 consecutive additional samples. This criterion was consistently satisfied at *N* = 50 for all experimental variants.

The width of the calibration window (the number of samples *N* for offset estimation) should be chosen so that the jitter is averaged out and so that Wi-Fi collisions causing at least one retransmission have time to occur, while at the same time not delaying the actual measurement excessively and also so that the window does not capture slow changes in the Wi-Fi conditions (which would cause the calibration to stop representing the pure clock offset). Through experiments (including, among others, an analysis of Wi-Fi retransmissions), the number *N* = 50 was selected (for a message transmission rate of 1 Hz), which gives a calibration time of 50 s. At higher message transmission rates, there is a greater probability of Wi-Fi collisions occurring, so the calibration time can be shortened (e.g., to 10–20 s at 10 Hz), which would give a number of calibration samples *N* in the range from 100 to 200. The experiments were carried out primarily under Windows, but rough trials performed under Linux gave similar results, with an indication of the possibility of slightly shortening the calibration window, which can be explained by the different operating characteristics of the task scheduler in the Windows and Linux systems.

In the developed application-layer subscriber software, an optional automatic determination of the calibration end point was also implemented, by analyzing the changes in the median value as a function of the number of samples *N*. For the selected value of *N*, this relationship should reach a plateau, and further increasing the number of samples should cause the median to change by less than 2 ms (since the aim of the calibration is to estimate the clock offset, of the order of tens of ms, rather than the network delay). Because in the Windows system the time may be automatically corrected in the background (this is related to the operation of the W32Time service), the offset may optionally (especially in the case of multi-hour measurements) be additionally refreshed “in the background” at certain intervals.

## 4. Results

Based on the data collected during the experiments, the following characteristics describing the quality of communication between the IoT_DUT device and the MQTT server were determined:

- The cumulative distribution function (CDF) of the message delivery delay;
- Jitter—the dispersion of the delay values;
- The message delivery effectiveness (PDR—packet delivery ratio);
- The stability of the message stream: goodput over time (moving average).

**Figure 3:**
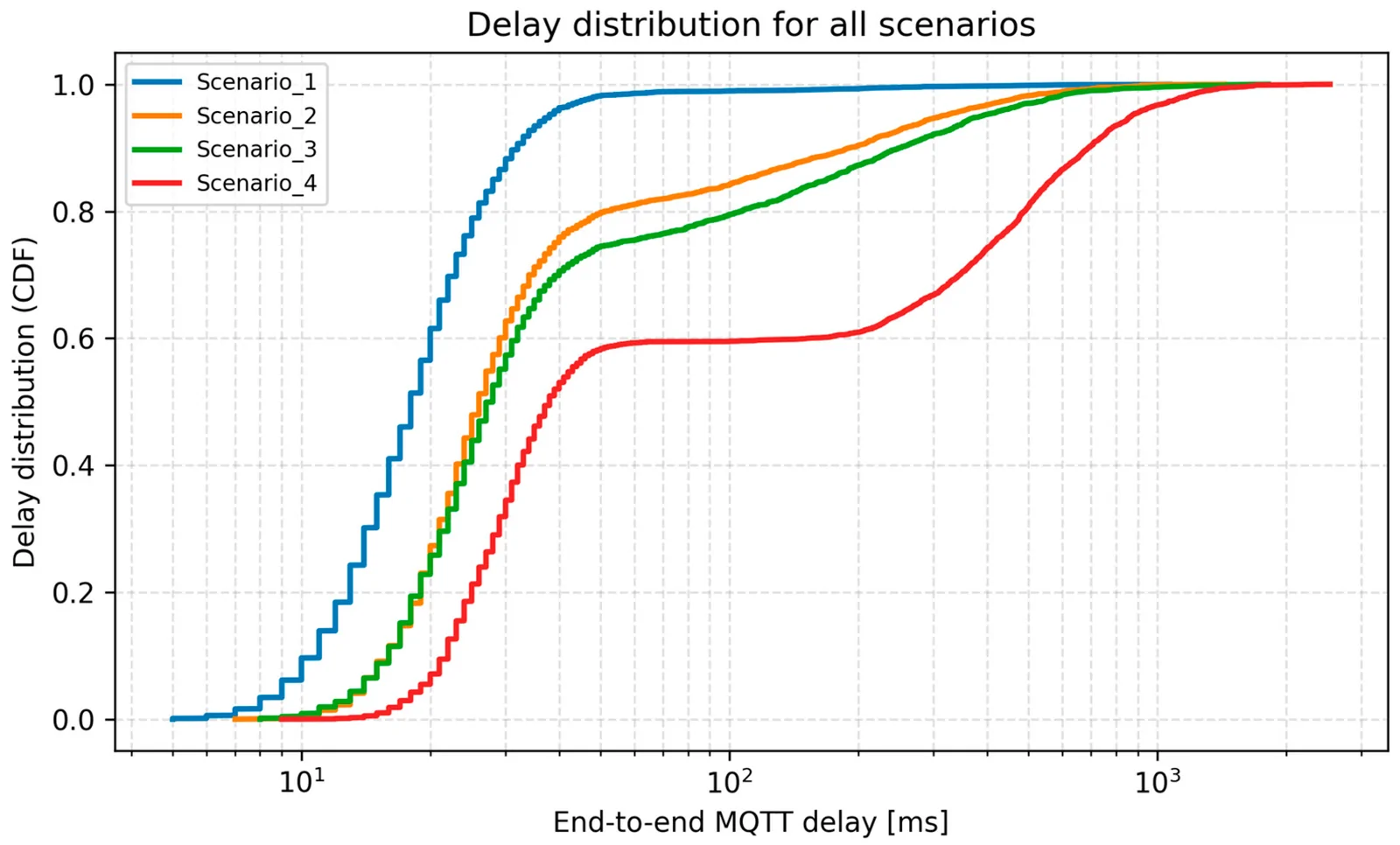
Empirical cumulative distribution function (CDF) of the MQTT delay for all scenarios.

Figure 3 presents the empirical cumulative distribution functions (CDFs) of the end-to-end delay of the MQTT transmission for all the analyzed scenarios.

**Figure 4:**
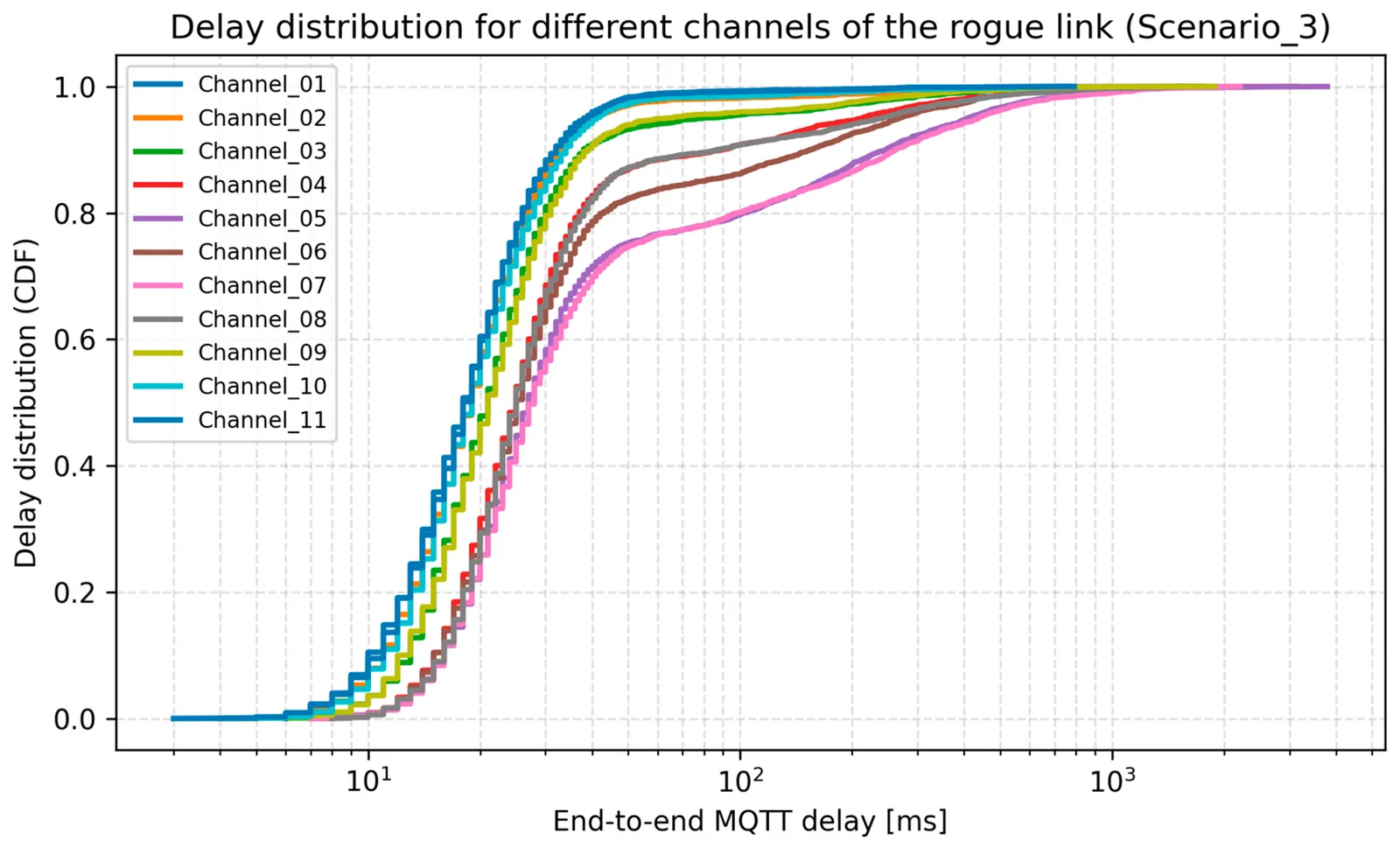
MQTT delay distribution functions for different channels of the interfering link in Scenario_3.

For the plots in Figure 3, the channel variants characterized by the strongest interference were selected. The selection was made on the basis of the CDFs determined for all tested channel variants and the selected variant maximized the P95 OWD relative to the reference scenario (Scenario_1). An example plot of the CDF for Scenario_3 is shown in Figure 4.

It follows from the plots in Figure 4 that the strongest interference occurred when the interfering link operated on the channels adjacent (i.e., channels 5 and 7) to the channel of the IoT_DUT device (channel 6). Therefore, one of these adjacent channels was selected for creating Figure 3, which covers all the scenarios.

The CDF plot makes it possible to assess what fraction of messages exhibits a delay smaller than a given value. In the scenarios with interference (Scenario_2–Scenario_4), the curves shift to the right and broaden, developing a longer tail, which indicates a more frequent occurrence of extreme delays and their greater variability. In the reference scenario (Scenario_1), most of the messages (over 95%) were delivered with a delay not exceeding 30 ms, and the delay distribution exhibited a relatively short tail. With the appearance of the interfering Wi-Fi 6 IEEE 802.11ax network (Scenario_2), a rightward shift of the CDF curve was observed, indicating an increase in the median and in the delay percentiles. A similar, though somewhat stronger, effect occurred in Scenario_3 (an interfering Wi-Fi 7, 802.11be network).

The most unfavorable conditions were recorded in Scenario_4. In this case, a pronounced lengthening of the distribution tail was observed, with individual samples reaching delays on the order of thousands of milliseconds. This phenomenon can be attributed to intensified frame retransmissions at the MAC level and to the backoff mechanisms arising from the contention of multiple networks for the same radio medium.

**Figure 5:**
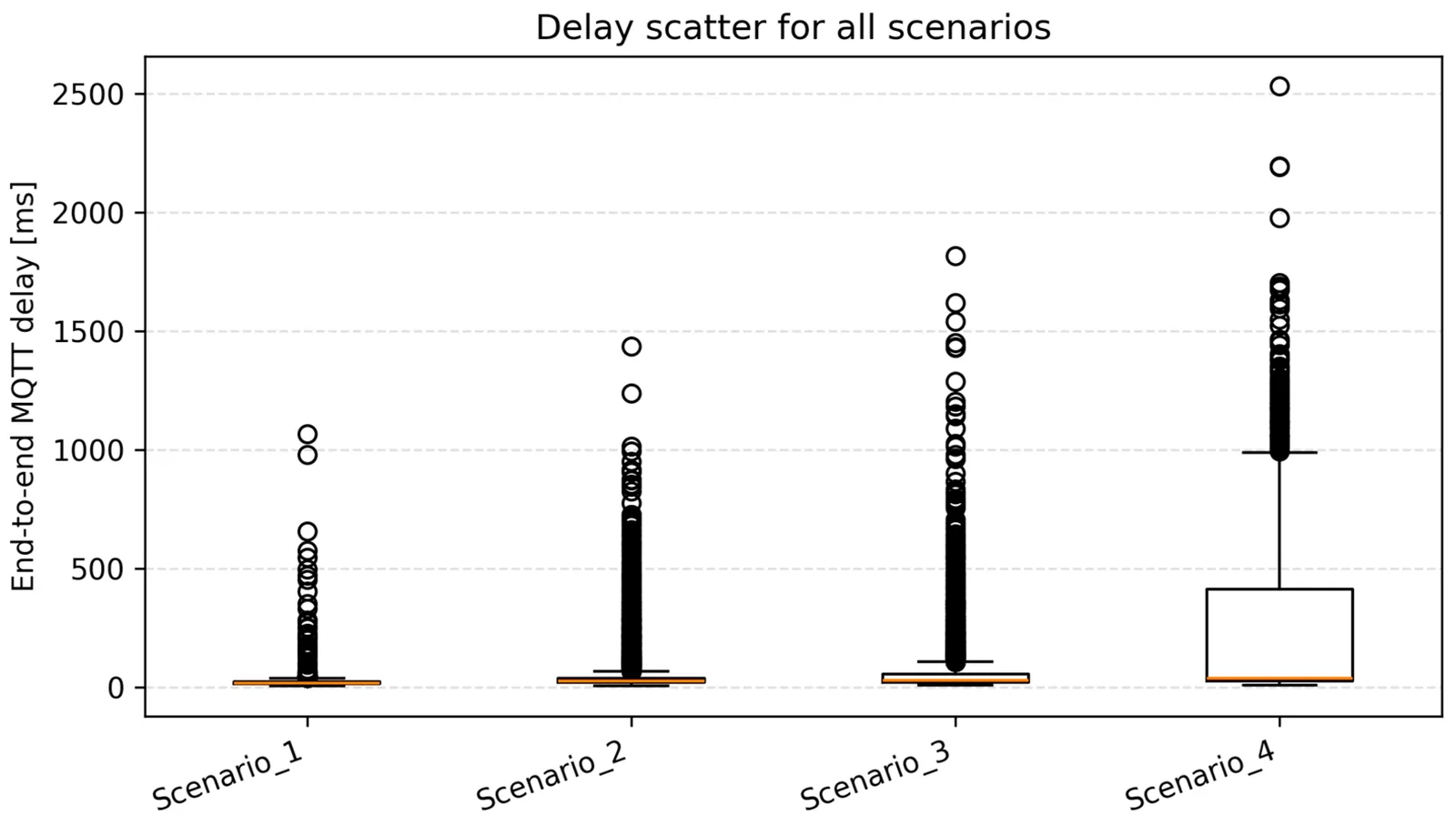
Dispersion of the MQTT delays for the studied scenarios.

Figure 5 presents the dispersion of the delays for the individual scenarios in the form of a box plot.

The plot in Figure 5 confirms the observation that the scenarios with interference have a median similar to that of the scenario with only the IoT_DUT device present but clearly longer tails and greater delay variability.

**Figure 6:**
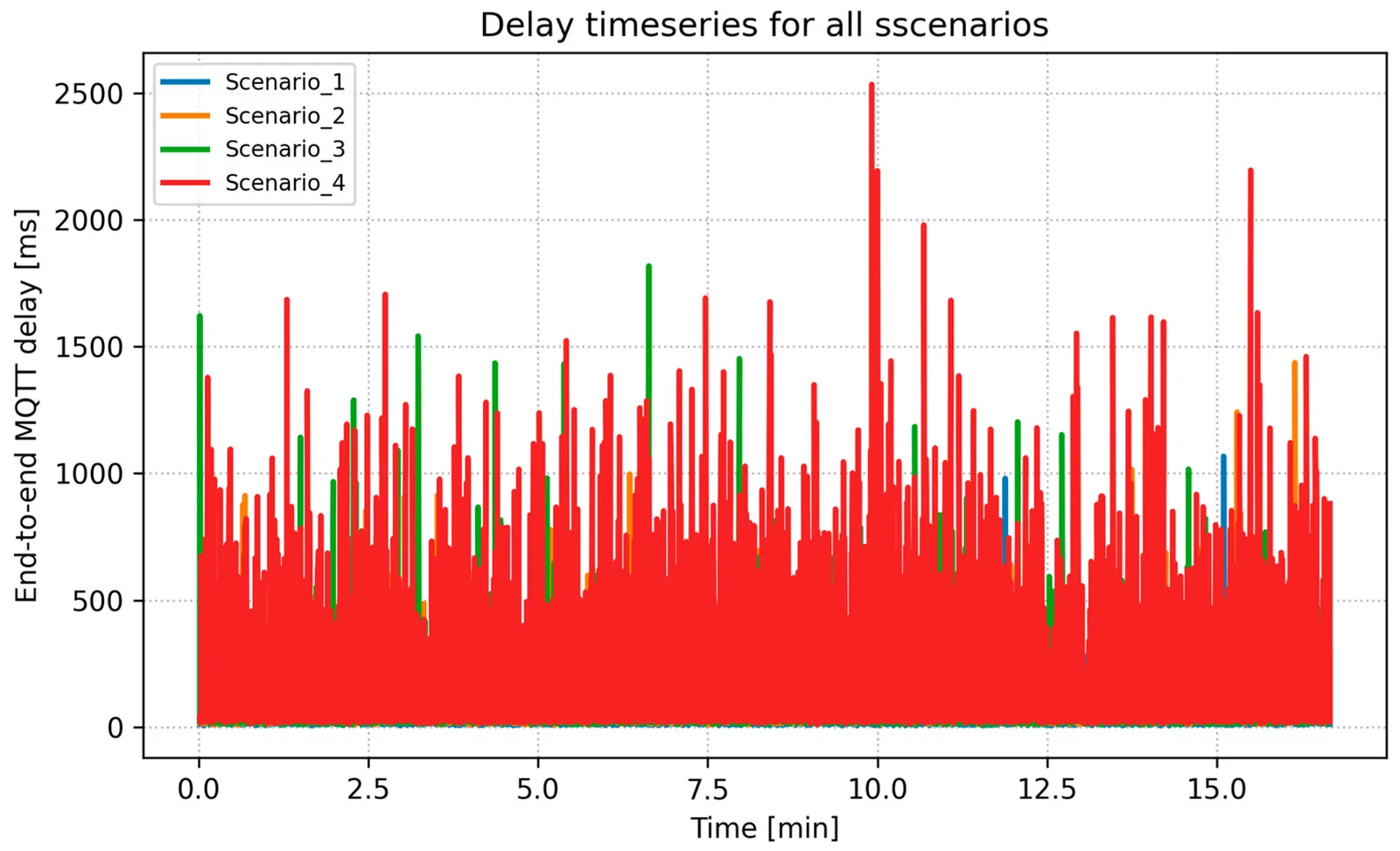
Time course of the delays for the studied scenarios.

Figure 6 presents the plot of the delays over time for the studied scenarios (the variants with the strongest interference were selected, analogously to the case of Figure 3).

Figure 6 illustrates the episodic peaks (the tails of the distribution) as a manifestation of effects occurring in the MAC layer (e.g., long aggregated transmissions, a series of backoffs, or retransmissions) in cases of the coexistence of multiple links operating in the same area.

**Figure 7:**
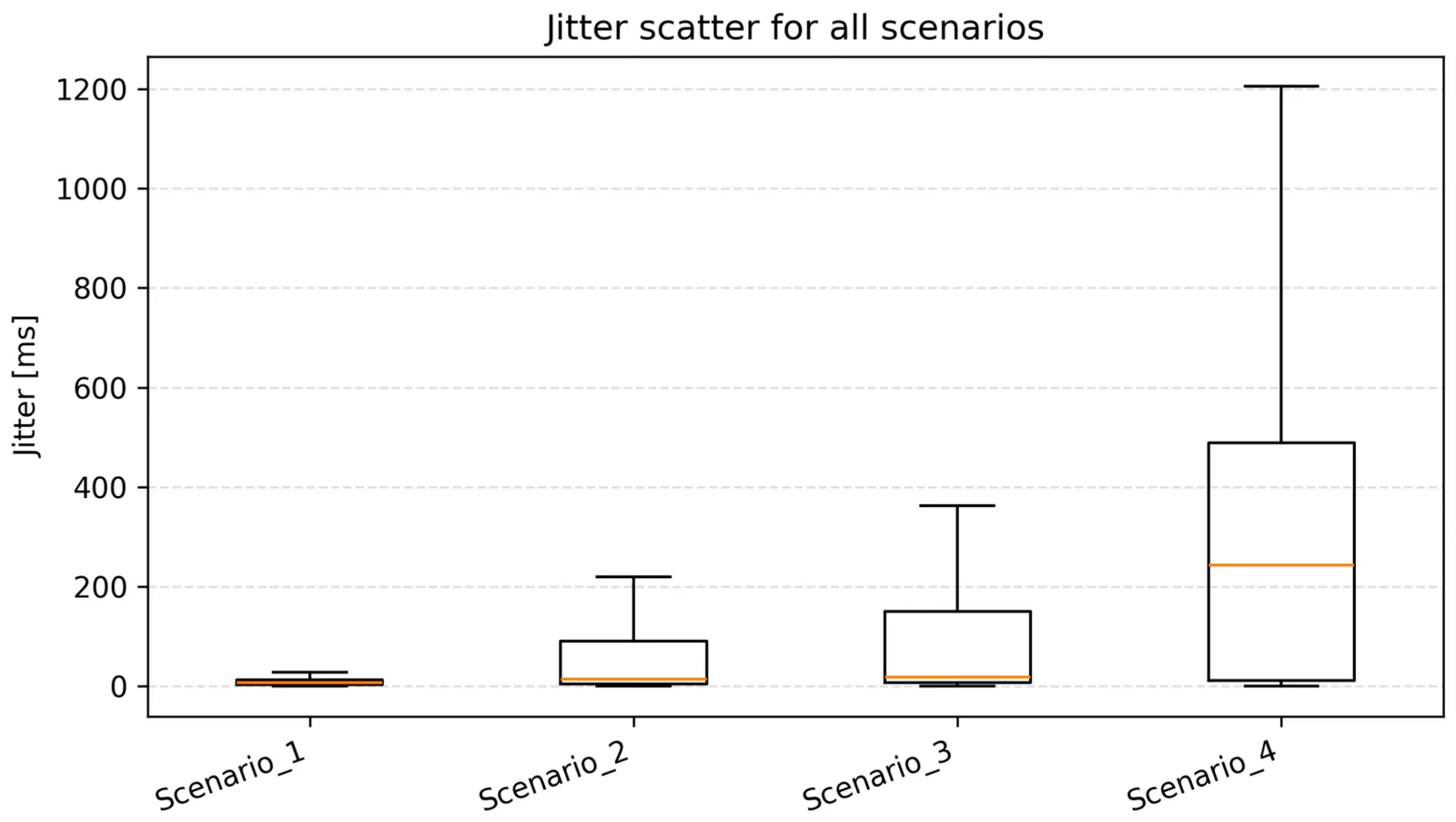
Dispersion of the jitter for the studied scenarios.

Figure 7 presents the distribution of the jitter, defined as the absolute value of the difference between successive delay measurements (Equation (2)).

In the reference environment (Scenario_1), the jitter remained at a low level, which testifies to the stable access of the IoT device to the transmission medium. In Scenarios_2 and _3, a noticeable increase in the median jitter and a widening of the interquartile range can be observed, which indicates an increased variability of the channel access times. In Scenario_4, a significant increase in both the median jitter and the extreme values was observed. Such a jitter characteristic is particularly unfavorable for IoT applications requiring regular data acquisition (e.g., control telemetry, quasi-real-time operation) or time synchronization, even if the average delay appears acceptable.

**Figure 8:**
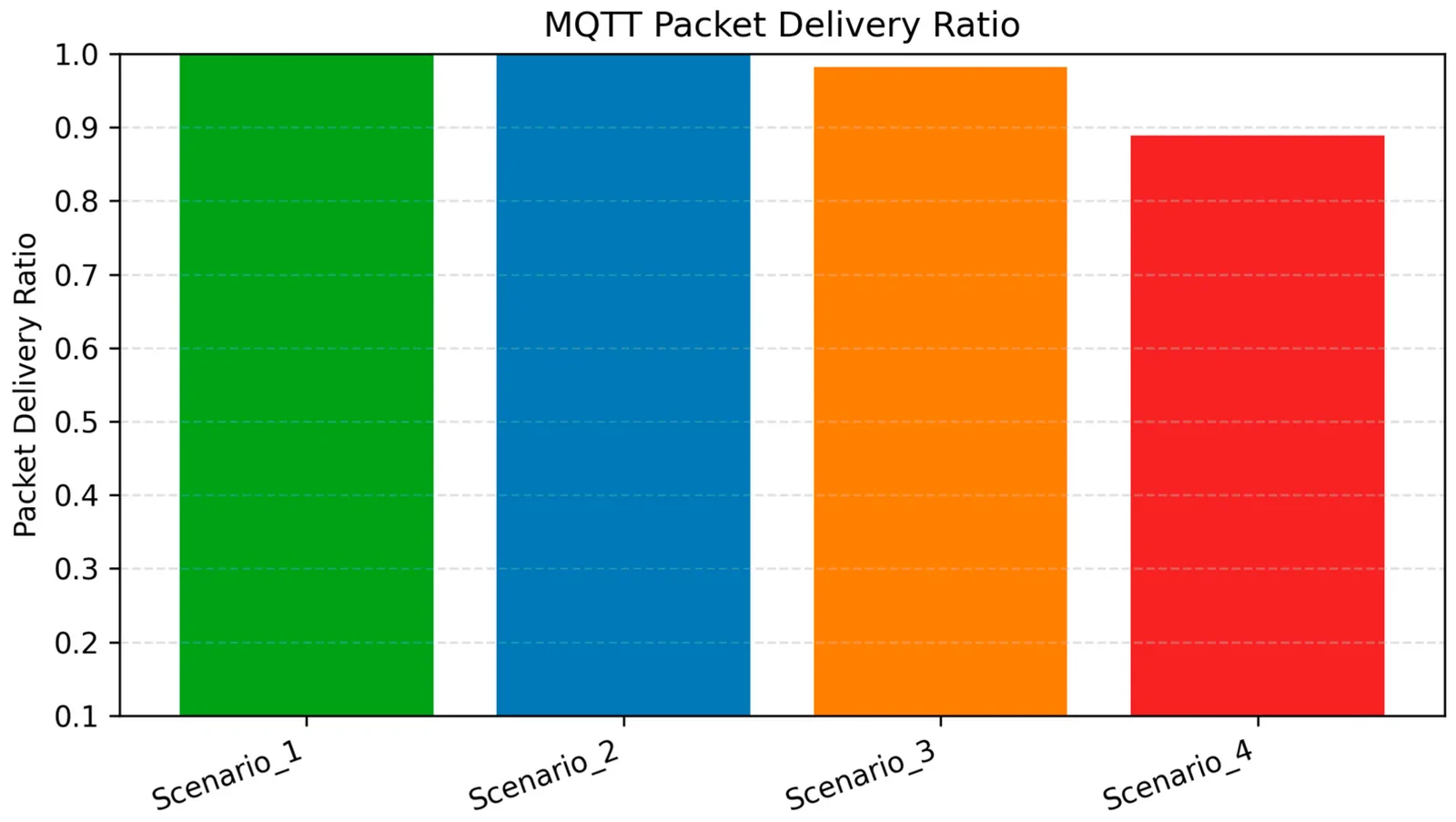
Message delivery effectiveness ratio in the studied scenarios.

Figure 8 presents the MQTT message delivery effectiveness ratio (PDR—packet delivery ratio). The PDR makes it possible to assess the stability of the communication. A decrease in the PDR with increasing density is a typical consequence of the growth in collisions/retransmissions and of medium congestion.

In the reference scenario (Scenario_1), the PDR was 100%, which confirms the correct and stable operation of the MQTT transmission in the 802.11n network in the absence of significant external interference. In the presence of the 802.11be network (Scenario_3), a slight decrease in the PDR was recorded, yet it still remained at a level acceptable for most telemetry applications. A significant decrease in the PDR was observed only in Scenario_4, where the increased network density caused more frequent message losses. Their causes were both retransmissions at the MAC level (greater contention for medium access) and timeouts while waiting for the acknowledgement of a Wi-Fi frame.

**Figure 9:**
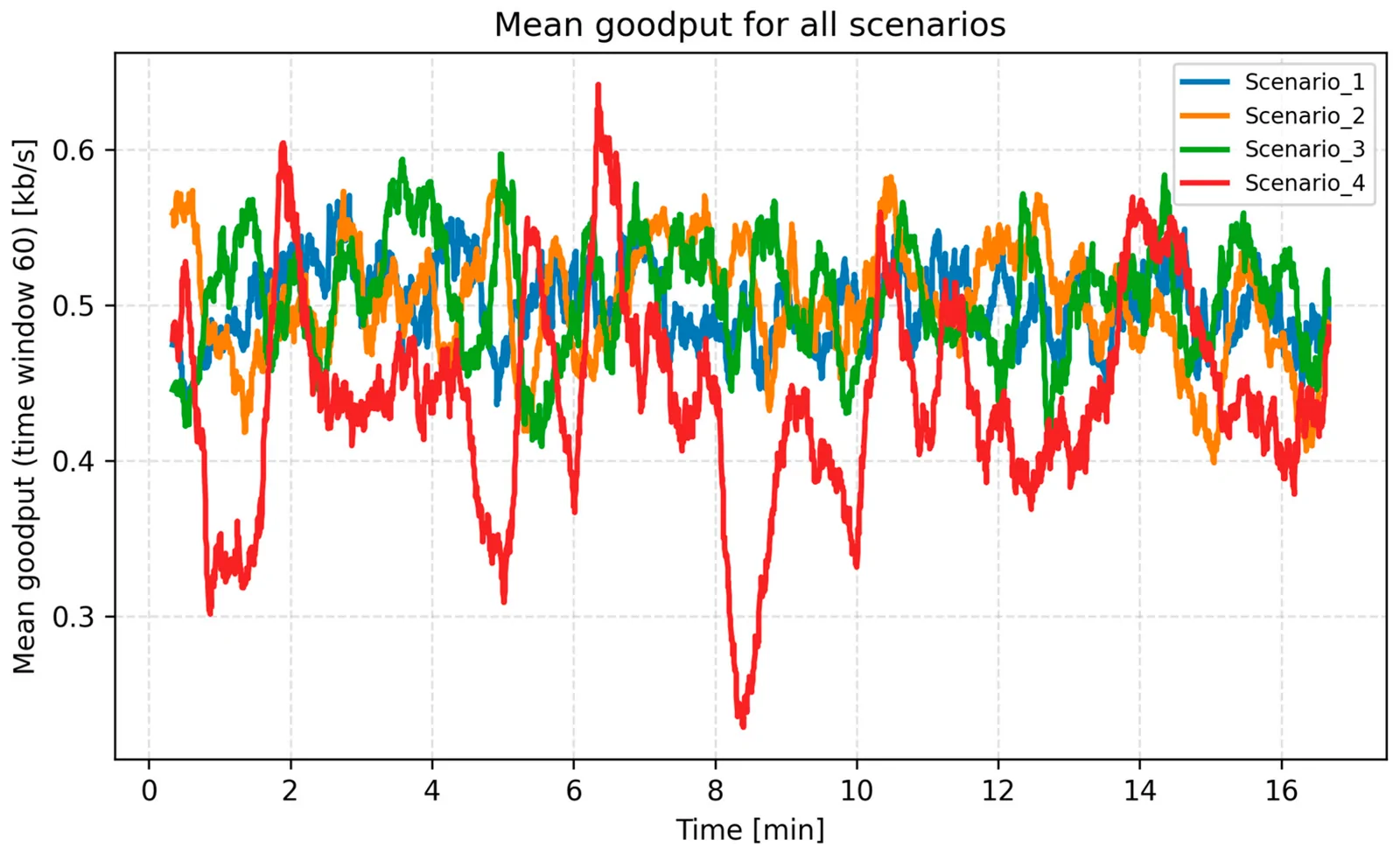
Application throughput over time (moving average over a 60 s window).

Figure 9 presents the time course of the effective application throughput (goodput), determined as a moving average over a time window of 60 s in width. The corresponding graphs for the 30 s and 120 s windows are provided in the Supplementary Materials.

In Scenarios_1–3, the goodput remained relatively constant, despite periodic fluctuations resulting from retransmissions and momentary collisions. In Scenario_4, however, more frequent throughput drops are noticeable, corresponding to periods with an increased number of message losses and delays. This indicates that, under conditions of high Wi-Fi network density in the 2.4 GHz band, even a modest MQTT data stream may be subject to significant quality fluctuations.

Table 3 summarizes the quality metrics computed from the experimental data for all four measurement scenarios, together with their confidence intervals (CIs).

Since the OWD and jitter distributions are heavily right-skewed with long tails, as visible in the CDF plots (Figure 3) and box plots (Figure 5 and Figure 7), classical parametric confidence intervals based on the normal distribution are not appropriate. The following methods have been applied instead, matched to the statistical nature of each metric:

- OWD and jitter: median and percentiles (non-parametric bootstrap CI). Bootstrap 95% confidence intervals (10,000 resamples, BCa method) were computed for the median, P95, and P99 of the OWD and jitter distributions for each scenario;
- PDR: Wilson score 95% confidence interval. For the PDR (a proportion), the Wilson score interval is used, which is valid for all sample sizes and does not require the normal approximation;
- Goodput: bootstrap CI for the mean. A bootstrap 95% confidence interval (10,000 resamples) for the mean goodput was computed for each scenario.

## 5. Discussion

### 5.1. Coexistence Effects of Wi-Fi Networks Operating in Different Generations of the IEEE 802.11 Standard

The effects observed in practice and presented in the previous section can be grouped into the issues discussed below, which are related to the features distinguishing modern Wi-Fi networks based on the IEEE 802.11ax (Wi-Fi 6) and IEEE 802.11be (Wi-Fi 7) standards from the older, yet still widely used (especially in IoT applications), IEEE 802.11n networks.

Throughout this section, statements are attributed to one of three evidential categories: (E) for findings directly supported by the experimental results of this study; (L) for explanations derived from the existing IEEE 802.11 literature and prior coexistence studies; and (I) for the author’s interpretations combining experimental observations with literature knowledge. This distinction is essential because the MAC-layer mechanisms cited as causal factors (OFDMA scheduling, Trigger frame signaling, and MAC retransmissions) were not directly instrumented in this study. Thus, the experimental evidence is limited to their application-layer consequences.

#### 5.1.1. A Paradigm Shift in Access to the Radio Medium

The results of this study demonstrate (E) that the presence of IEEE 802.11ax and IEEE 802.11be networks in the 2.4 GHz band leads to a systematic increase in the one-way MQTT delay, jitter, and delay tail of the IEEE 802.11n IoT device, with the effect most pronounced under dual-interference conditions (Scenario_4). These observations are consistent with (L) a fundamental difference in the medium-access design philosophy of the IEEE 802.11n and the newer high-efficiency (HE) standards, such as IEEE 802.11ax and IEEE 802.11be. The 802.11n standard was designed in an era when the number of active stations in a single radio area was relatively small, and the main objective was to increase point-to-point throughput, for example, through the MIMO technique and frame aggregation. In the 802.11n standard [5] (L), medium access relies exclusively on the random CSMA/CA mechanism, designed for low-station-density environments, in which each station independently competes for channel access using exponential binary backoff, which under conditions of high contention leads to random and unpredictable delays.

In contrast, the IEEE 802.11ax standard [6] introduces organized uplink transmission scheduling via OFDMA and Trigger frames, through which the access point allocates specific resource units (RUs) to individual stations. According to the survey [8] (L), these mechanisms substantially improve spectral efficiency and reduce access latency variance for HE stations in dense environments. However, the results of the conducted experiments show that the improvement in system efficiency in the new Wi-Fi standards is not neutral for legacy devices, which are still in common use and, in the case of IoT applications, may even be regarded as dominant. The interpretation that HE scheduling creates an effective medium-access asymmetry disadvantageous to legacy CSMA/CA devices is the author’s own interpretation (I), consistent with the coexistence analysis in [13,14] (L). Since OFDMA scheduling and Trigger frame parameters were not directly measured, this interpretation is not experimentally verified within this study.

From an RF perspective (L/I), the channel-by-channel analysis of Figure 2 and Figure 4 implicitly captures the effect of the adjacent channel interference ratio (ACIR), which combines the adjacent channel leakage ratio (ACLR) of the high-efficiency (HE) interferer’s transmitter and the Adjacent Channel Selectivity (ACS) of the 802.11n IoT device’s receiver. The spectral masks shown in Figure 2 represent the ACLR-equivalent constraint for IEEE 802.11 transmitters: as channel separation between the DUT and the interferer decreases, both the spectral overlap and the effective interference power within the DUT’s channel increase, resulting in a lower effective ACIR. This RF-layer effect is reflected in the application-layer results: the strongest MQTT delay degradation is observed for the minimum-separation (adjacent-channel) configurations (channels 5 and 7 relative to the DUT’s channel 6), where the ACIR is at its minimum, while the degradation diminishes systematically as channel separation increases and the ACIR improves.

#### 5.1.2. Asymmetry of Medium Access in a Mixed Environment

The experimental results indicate (E) an asymmetry in the effective quality of medium access between the IEEE 802.11n IoT device and the HE stations operating in the same environment. Despite the formal preservation of backward compatibility at the PHY and MAC layers, as required by the IEEE 802.11ax standard [6] (L), the 802.11n device is unable to participate in OFDMA resource allocation or Trigger-frame-based scheduling. According to the standard [6] and the coexistence analysis in [14] (L), HE stations benefit from organized transmission opportunities coordinated by the access point, while legacy 802.11n devices continue to compete for channel access using random CSMA/CA.

Since MAC-layer scheduling parameters, including Trigger frame intervals, TXOP durations, and per-station backoff distributions, were not directly measured, the following mechanistic explanation is based on the existing literature rather than on direct experimental evidence (L/I): 802.11n stations are likely to experience extended backoff phases during periods of intensive HE transmission, because HE stations operating in scheduled bursts occupy the channel for extended periods without presenting random contention opportunities, thereby reducing the effective channel access probability for CSMA/CA-dependent devices. The experimental evidence consistent with this interpretation (E) is: (i) the systematic increase in jitter and the lengthening of the tail of the delay distribution observed in Scenarios_2 and _3 compared to Scenario_1 and (ii) the channel-dependent degradation pattern of Figure 4, which follows the spectral overlap analysis of Figure 2 and cannot be attributed to channel-independent artefacts such as software delays or measurement jitter.

#### 5.1.3. Extreme Delays and the Determinism of IoT Communication

Although the average delay values in Scenarios_2 and _3 do not always deteriorate strongly (E), the analysis of the CDF distributions reveals a significant increase in the extreme values (the P95 and P99 percentiles). From the perspective of IoT applications, this is not a negligible phenomenon, and the following examples illustrate the significance of extreme episodic delays in representative application domains (L):

- Industrial automation and SCADA: control-loop periods of 10–100 ms are typical in process automation; episodic delays exceeding this threshold can cause false timeout alarms and may trigger unnecessary emergency shutdown procedures [32].
- Distributed sensor fusion and data acquisition: irregular message delivery times disrupt the temporal alignment of measurements from multiple sensors, degrading the accuracy of fused data products and event-detection algorithms [33].
- Medical IoT (remote patient monitoring): unpredictable delivery latency may compromise the reliability of automated clinical alerts, where regulatory frameworks impose strict maximum latency requirements.
- MQTT session management: if the application-layer delay exceeds the configured MQTT keep-alive interval, the broker closes the connection, triggering a TCP/MQTT reconnection sequence and potential message loss [15,16].

These examples, drawn from the literature (L), illustrate that the fundamental problem revealed by the experiments (E) is not the average throughput but the loss of delay determinism in an environment dominated by HE networks. Even sporadic delays on the order of hundreds of milliseconds can cause an accumulation of buffered events in the higher-level application, false alarms driven by timeouts, and forced re-establishment of MQTT/TCP connections, which are consequences not visible with PDR-based monitoring alone.

#### 5.1.4. Packet Delivery Ratio vs. Quality Stability

A notable observation (E) is that the PDR remains relatively high even in the presence of the 802.11ax and 802.11be networks (though not simultaneously, which might superficially suggest acceptable system operation. However, putting together the PDR with the analysis of jitter and of goodput over time reveals a significant difference between delivery and the quality of delivery.

Although MAC-layer retransmission counters were not logged in this study, the coexistence of a high PDR with a substantially lengthened tail of the delay distribution and elevated jitter is quantitatively consistent with the retransmission compensation mechanism of IEEE 802.11n (L/I): the standard specifies up to seven retransmission attempts per frame with exponential binary backoff, reaching a maximum contention window of 1023 slots [5] (L). The resulting theoretical maximum accumulated backoff (~143 ms at 20 µs/slot) and TCP retransmission timeout are consistent with the delay spike magnitudes observed in Figure 6 (100–2500 ms). Similar behavior has been reported for wireless MQTT systems in [21,22] (L). This interpretation implies that MAC and TCP retransmission mechanisms recover most lost frames, but at the cost of

- An increase in one-way delay;
- An increase in temporal variability (jitter);
- A temporary blocking of the application-layer callback.

As a consequence (E), what is observed is not primarily a decrease in average throughput but an increase in delay variability, manifesting as elevated jitter and heavy-tailed OWD distributions. A high PDR may therefore mask genuine quality problems that become apparent only in the temporal analysis—a finding consistent with the literature on IoT protocol performance [21,22,32] (L).

#### 5.1.5. Summary of the Legacy vs. High-Efficiency Wi-Fi Discussion

In a mixed environment, HE stations (802.11ax/802.11be) benefit from efficiency-enhancing mechanisms (L) [6,7], while legacy 802.11n devices remain dependent on the traditional stochastic CSMA/CA mechanism. The experimental results of this study confirm (E) that this configuration leads to an increase in jitter and in the tails of the delay distribution, even when the PDR remains relatively high, since retransmissions compensate for packet losses at the cost of delay.

Table 4 presents a structured comparison of legacy and HE Wi-Fi mechanisms and their consequences for MQTT communication quality. Each row is annotated with its evidential basis: (E) directly supported by the experiments in this paper; (L) based on IEEE 802.11 literature or prior coexistence studies; and (I) the author’s interpretation. Rows marked (L) or (I) represent mechanism-level explanations rather than directly verified findings of this study.

In summary, the results of this study suggest (E/I) that high-efficiency Wi-Fi standards fulfil their primary objective of maximizing radio-medium utilization in dense environments. However, in heterogeneous deployments this is achieved partly at the expense of legacy devices that are unable to participate in the new transmission-coordination mechanisms. For IoT systems, this means that continued reliance on IEEE 802.11n in the 2.4 GHz band may lead to quality problems that are difficult to detect and are invisible when analyzing only the average parameters yet crucial from the perspective of temporal stability and near-real-time application performance and reliability of the entire system.

The above summary is supported by the statistical parameters (especially confidence intervals) presented in Table 3. Although the median OWD values are similar across Scenarios_1–3 (18, 26, and 28 ms, respectively), the distribution tail expands dramatically with increasing interference density: the P95 OWD increases 23-fold from 38 ms [36–38 ms] in the reference scenario to 869 ms [842–921 ms] in Scenario_4, and the P99 OWD reaches 1287 ms [1226–1414 ms]. All pairwise differences are statistically significant at *p* < 0.001 (Wilcoxon rank-sum test). Jitter is the most severely affected metric, with the median increasing 35-fold from 7 ms to 247 ms [227–266 ms] and P95 jitter reaching 931 ms [887–987 ms] in Scenario_4. Notably, PDR remains above 99.7% and statistically indistinguishable across Scenarios_1–3 (S1 vs. S3: χ^2^ = 0.25, *p* = 0.617), despite the substantial degradation of temporal parameters, confirming that MAC- and TCP-layer retransmissions mask genuine quality problems by compensating for packet losses at the cost of increased delay. The PDR falls significantly only in Scenario_4, to 88.9% [87.7–90.0%] (χ^2^ = 331, *p* < 10^−73^). Mean goodput remains stable across Scenarios_1–3 (~0.500–0.505 kbps) and decreases significantly only in Scenario_4 to 0.443 kbps [0.434–0.453 kbps]. These results demonstrate that coexistence degradation manifests primarily as a loss of temporal determinism and that the PDR alone is insufficient to characterize MQTT communication quality in mixed-standard Wi-Fi environments (E) + (I).

### 5.2. Implications for Standards and Engineering Practice

#### 5.2.1. Conclusions for the Design and Evolution of the IEEE 802.11 Standards

The results obtained in this study suggest (E/I) that the era of homogeneous WLAN networks is gradually ending and that IoT systems increasingly operate as “guests” in environments dominated by high-throughput traffic. This forces a change in approach both in hardware design and in the planning of network infrastructure. The results obtained highlight the practical limitations of the backward-compatibility mechanism used in the Wi-Fi standards. Although the IEEE 802.11ax and 802.11be standards formally ensure interoperability with legacy devices, the experimental analysis of transmission quality indicates that functional compatibility does not guarantee an equivalent quality of service. This finding is consistent with the literature on mixed 802.11ax/legacy environments [13,14] (L).

From the perspective of future revisions of the IEEE 802.11 standards, the results of this study suggest (I) consideration of the following:

- Mechanisms for cross-generation fairness of channel access, ensuring that legacy CSMA/CA devices are not systematically disadvantaged during periods of high HE load;
- Explicit differentiation and protection of low-throughput and time-critical traffic (typical of IoT and telemetry applications);
- Improved reporting and control of the impact of OFDMA scheduling and Trigger frame mechanisms on legacy station performance.

The absence of such mechanisms may lead to a situation in which formally compatible devices do not meet the quality requirements of the application. These suggestions are based on experimental observations and on the limitations of backward compatibility identified in the literature [13,14] (L/I). It is important to emphasize that the experiments were conducted under specific controlled conditions (one IoT device, one or two fully loaded HE interferers, and a 2.4 GHz band) and that generalization to other deployment scenarios requires further experimental validation.

#### 5.2.2. Recommendations for IoT System Designers

The research results carry several important practical recommendations for engineers designing IoT systems. It should be emphasized that the following engineering recommendations are derived from the specific experimental conditions studied in this paper: an IEEE 802.11n IoT device operating at 1 Hz, QoS 0, and 256 B payload in a 2.4 GHz band shared with at most two HE-generation interferers operating at full load. Generalization to other traffic profiles, device densities, or interference levels requires additional experimental validation:

1. Evaluating not only the PDR but also the jitter and the delay percentiles (E). The classical reliability indicators (PDR) may mask significant temporal quality problems, as demonstrated by the simultaneous occurrence of a high PDR and substantially lengthened tails of OWD distribution in Scenarios_2 and _3. Analysis of the P95/P99 OWD percentiles and median jitter should be regarded as standard practice in the validation and acceptance testing of IoT systems.
2. Avoiding the congested 2.4 GHz band in modern WLAN environments and taking into account the limitations of adaptive RF band mechanisms for legacy IoT devices (L/I).

In environments dominated by 802.11ax/be networks, the 2.4 GHz band ceases to be a natural choice for 802.11n devices. Modern Wi-Fi infrastructure supports several mechanisms for adaptive spectrum and band management, including Automatic Channel Selection (ACS), IEEE 802.11k/v-based band steering, Dynamic Frequency Selection (DFS) in the 5 GHz band, IEEE 802.11ax BSS coloring, and Wi-Fi 7 Multi-Link Operation (MLO). In principle, these mechanisms could partially alleviate coexistence issues in mixed-standard environments. However, in the context of legacy IEEE 802.11n IoT devices of the class studied in this paper, their applicability is severely constrained:

- Low-cost IoT microcontrollers (including the ESP32 DevkitC-32E used in this study) are typically single-band 2.4 GHz devices, physically incapable of band switching regardless of interference level; band steering (802.11v) is therefore ineffective for this device class.
- IEEE 802.11k/v client-side support is absent from most lightweight IoT Wi-Fi firmware stacks, which prioritize minimizing RAM/ROM footprint over full IEEE 802.11 feature compliance.
- In practice, the channel of the IoT access point is typically statically configured by the network administrator to ensure predictable, stable connectivity; dynamic AP channel reassignment would disrupt ongoing MQTT/TCP sessions and trigger reconnection sequences.

Consequently, the coexistence degradation documented in this paper largely cannot be autonomously mitigated by legacy 802.11n IoT devices or their dedicated access points. Adaptive channel management mechanisms provide relief primarily for modern, dual-band, full-featured client stations but not for the resource-constrained, single-band end-devices that are the subject of this study (I). This observation reinforces the practical significance of the findings: quantifying the impact of HE-network coexistence on legacy IoT devices is essential precisely because these devices cannot autonomously avoid it.

3. Considering segmentation of the radio and logical network structure (I).

Providing dedicated access points for IoT traffic, limiting channel widths in HE networks sharing the 2.4 GHz band, and implementing VLAN/SSID separation for legacy devices can partially reduce interference without requiring hardware replacement.

4. Plan for technological migration rather than treating it as a future option (I).

Long-term IoT projects should take migration into account:

- To newer Wi-Fi standards;
- To the less congested 5 GHz or 6 GHz band;
- To alternative technologies (Ethernet, sub-GHz, and LPWAN) if temporal determinism is critical.

It is recognized, however, that in practice such migration may be significantly con-strained by the economic cost of hardware replacement, the integration complexity of existing embedded systems, the long operational lifetimes typical of industrial and building IoT deployments, and applicable regulatory limitations on frequency bands.

#### 5.2.3. Significance for Audits and Acceptance Tests of IoT Systems

In the context of industrial and building deployments (like smart building and critical infrastructure), the results of this study suggest (E/I) the need for a revised approach to IoT system acceptance testing. Tests performed under laboratory conditions or in homogeneous networks may not reflect the actual operating conditions of the system deployed in a modern, heterogeneous 2.4 GHz Wi-Fi environment.

The acceptance testing procedure proposed in this section represents the author’s own engineering recommendation, derived from the experimental findings of this study. It is not directly based on, nor formally compliant with, existing IEEE standards (such as IEEE 802.11k/v/r) or industry certification frameworks (such as Wi-Fi Alliance certification programs or IEC 62443 for industrial security), although it is consistent with their general quality-assurance principles. In practice, this procedure could be implemented as follows and requires only commercially available equipment (Wi-Fi 6/7 access points, a laptop running iPerf3 and the measurement software described in Section 2):

- Tests performed in the presence of HE (802.11ax/be) networks operating on the same channel and on the minimum-separation adjacent channels (±1 channel);
- Long-term measurements of OWD, jitter and especially extreme delays;
- Analysis of the stability of application-layer protocols (MQTT and CoAP) with respect to keep-alive timeouts and session reconnection frequency under representative coexistence conditions.

Such a procedure would allow system integrators and auditors to assess whether an IoT deployment maintains its temporal quality requirements: not only in the clean-room conditions of initial commissioning but in the increasingly heterogeneous 2.4 GHz environments characteristic of modern buildings and industrial facilities.

## 6. Conclusions

The article focuses on the impact of modern Wi-Fi networks (802.11ax/802.11be) on IoT devices employing 802.11n connectivity and MQTT in the ISM band (2.4 GHz). The conducted experiments confirm that the coexistence of IEEE 802.11ax and IEEE 802.11be networks in the 2.4 GHz band can significantly degrade the temporal transmission characteristics of MQTT traffic generated by IEEE 802.11n devices. This degradation mechanism is consistent with the asymmetry of medium access in mixed-standard environments, as described in the existing literature [13,14]: IEEE 802.11ax/be stations benefit from organized uplink scheduling (OFDMA and Trigger frames), while IEEE 802.11n devices remain constrained to contention-based random access via CSMA/CA, resulting in increased backoff durations and retransmission probabilities. The author notes that these causal mechanisms were not directly measured in this study and their attribution relies on the existing literature author’s interpretations, combining experimental observations with literature knowledge.

The most strongly observed effect is the increase in jitter and the occurrence of extreme delays (manifested as a heavy tail in the delay distribution), which limit the determinism of IoT communication. Packet delivery ratio (PDR) degradation is typically less severe than the deterioration of temporal parameters, suggesting that MAC- and TCP-layer retransmission mechanisms compensate for packet losses at the expense of increased latency. Consequently, the assessment of IoT communication quality in a mixed environment should take into account full delay distributions and jitter characteristics, rather than relying solely on the mean values and the reliability of packet delivery.

To the best of the author’s knowledge, this is the first unique experimental study providing direct, application-layer measurements of MQTT communication quality (OWD, jitter, PDR, and goodput) for an IEEE 802.11n IoT device coexisting with both IEEE 802.11ax (Wi-Fi 6) and IEEE 802.11be (Wi-Fi 7) networks in the 2.4 GHz band. Unlike previous work, which focuses on MAC/PHY metrics, simulation-based analysis, or the 5 GHz band, this study targets the application-layer perspective of a representative legacy IoT end-device under realistic coexistence conditions. The results obtained indicate the following:

- The presence of modern IEEE 802.11ax and 802.11be networks in the 2.4 GHz band degrades the temporal parameters of MQTT transmissions carried out by 802.11n devices.
- The greatest impact is observed in the area of
  - Jitter—the median jitter increases from 7 ms in Scenario_1 to 247 ms in Scenario_4;
  - Extreme delay values: P95 OWD increases from 38 ms in Scenario_1 to 839 ms in Scenario_4.
- The decrease in message delivery effectiveness (PDR) becomes significant only at a high density of networks (the PDR is practically 100% in Scenarios_1–3 and decreases to 89% in Scenario_4).
- Even when a high PDR is maintained, the stability of the data stream may be limited.

The experimental results obtained make it possible to identify the practical implications for the design and operation of IoT systems (apply to the experimental conditions considered in this study):

- The 2.4 GHz band ceases to be a “safe choice” for legacy devices, especially in environments with a high density of modern Wi-Fi networks;
- Backward compatibility does not imply an equivalent quality of service—802.11n devices formally operate correctly but experience a degradation of their temporal parameters;
- Radio planning becomes critical: the choice of channel, limiting the channel widths in HE networks, and the segmentation of the radio space of the infrastructure (dedicated SSIDs/VLANs and channel planning) can partially mitigate the negative effects;
- For IoT applications requiring temporal predictability, the following may be necessary: migration to less congested bands (5 GHz/6 GHz), improved channel planning, reduction in co-channel interference, or the use of alternative technologies (e.g., wired, sub-GHz, LPWAN) depending on the requirements.

The main limitations of this study are as follows:

- The measurements were conducted in a single controlled indoor laboratory environment (LOS, ~70 m^2^);
- Only one IoT device model (ESP32 DevkitC-32E) and one access-point model (MikroTik hAP ac^2^) were used in the IoT link;
- A single MQTT traffic profile was tested (1 Hz publication rate, QoS 0, 256 B payload);
- MAC-layer statistics (per-frame retransmission counts, RSSI, MCS index) were not collected;
- The interfering networks operated at maximum load (full iPerf3 saturation), representing a worst-case interference scenario.

These limitations define the scope within which the conclusions are valid and motivate the directions for future work listed below:

- A comparison of the 2.4 GHz, 5 GHz, and 6 GHz bands for the same class of IoT devices;
- An analysis of the influence of MQTT application-layer parameters (QoS 0/1/2, payload size, publication frequency, and TLS protection) on delays and stability;
- Investigation of scenarios with a larger number of simultaneously active IoT devices and interferers;
- Extending the measurements with PHY/MAC metrics (MCS, number of retransmissions, channel utilization, and channel width) and their correlation with application-layer metrics in Windows and Linux systems;
- Studies in an environment with controlled interference (different load levels generated by HE stations);
- Tests with a real Wi-Fi signal in the background;
- Influence of IoT link to goodputs of Wi-Fi 6 and Wi-Fi 7 networks;
- An analysis of the influence of Wi-Fi 7 mechanisms (e.g., MLO, MU-MIMO) on the fairness of access for legacy IoT devices.

## Figures and Tables

**Table 1:** Parameters of the experimental setup configuration.

Parameter	Value/Details
IoT_DUT module	Espressif ESP32 DevkitC-32E
IoT_DUT Wi-Fi standard	IEEE 802.11n, 2.4 GHz, 20 MHz, STA mode
AP1 (DUT access point)	MikroTik hAP ac^2^, RouterOS 7.22.1
AP1 Wi-Fi standard	IEEE 802.11n, 2.4 GHz, ch. 6, 20 MHz, AP mode
AP2 (Rogue link 1)	MikroTik hAP ax^3^, RouterOS 7.22.1
AP2 Wi-Fi standard	IEEE 802.11ax, 2.4 GHz, 20 MHz
AP3 (Rogue link 2)	Ubiquiti U7 Pro Wall
AP3 Wi-Fi standard	IEEE 802.11be, 2.4 GHz, 20 MHz
AP3 firmware	UniFi firmware 8.5.21
MQTT broker	Eclipse Mosquitto 2.1.2
iPerf version	iPerf3 3.21
Room conditions	~70 m^2^, LOS conditions for all devices Links distances ~8 m

**Table 2:** Configuration of all experimental scenarios.

Scenario_1	Networks	Standard (s)	DUT Channel	Interferer Channel(s)	Variants	Samples Per Variant	Total Samples
Scenario_1	IoT_DUT only	802.11n	6	---	1	1800	1800
Scenario-2	IoT_DUT + 1 interferer	802.11n + 802.11ax	6	1–11	11	1800	19,800
Scenario_3	IoT_DUT + 1 interferer	802.11n + 802.11be	6	1–11	11	1800	19,800
Scenario_4	IoT_DUT + 2 interferers	802.11n + 802.11ax + 802.11be	6	26 channel pairs (see text)	26	1800	46,800

**Table 3:** Confidence intervals for all reported metrics and scenarios.

Metric	Scenario_1 (No Foreign Networks)	Scenario_2 (Wi-Fi 6)	Scenario_3 (Wi-Fi 7)	Scenario_4 (Wi-Fi 6 and Wi-Fi 7)
OWD Median [ms]	18 [18, 18]	26 [25, 25]	28 [27, 28]	38 [36, 38]
OWD P95 [ms]	38 [36, 38]	312 [286, 349]	390 [360, 429]	869 [842, 921]
OWD P99 [ms]	103 [64, 213]	618 [575, 711]	697 [640, 839]	1287 [1226, 1414]
OWD Max [ms]	1067	1436	1817	2532
Jitter Median [ms]	7 [7, 7]	14 [12, 14]	19 [16, 19]	247 [227, 266]
Jitter P95 [ms]	29 [26, 31]	410 [385, 439]	461 [440, 501]	931 [887, 987]
PDR [%]	99.77 [99.52, 99.89]	100.00 [99.87, 100.00]	99.70 [99.43, 99.84]	88.90 [87.73, 89.98]
Mean Goodput [kbps]	0.500 [0.493, 0.509]	0.502 [0.495, 0.509]	0.505 [0.497, 0.513]	0.443 [0.434, 0.453]

**Table 4:** Comparison of the impact of legacy Wi-Fi and high-efficiency Wi-Fi mechanisms on the MQTT transmission of IoT devices.

Area/Mechanism	Legacy Wi-Fi (IEEE 802.11n)	High-Efficiency Wi-Fi (IEEE 802.11ax/802.11be)	System-Level Consequences (Mixed Environment)	Experimental Observations (MQTT, 802.11n)	Evidential Basis
Medium-access model	Random CSMA/CA access	Scheduled access (OFDMA, trigger frames)	Uneven channel access for legacy stations	Increase in jitter and randomness of delays	(E) + (L) [5,6,7]
Transmission scheduling	No central coordination	Central scheduling by the access point	Preference for HE stations during periods of high load	Heavy-tailed OWD distribution	(I) + (L) [6,13,14]
Response to network densification	Decrease in efficiency with a larger number of stations	Maintenance of high system efficiency	System efficiency at the expense of legacy devices	Isolated delays in the order of hundreds of ms	(E) + (L) [8,13,14]
Frame aggregation	Limited (A-MPDU)	Strong aggregation and time scheduling	Longer occupation of the medium by HE frames	Momentary “starvation” of IoT transmissions	(I) + (L) [5,6]
Access fairness	Relative equality among stations of the same generation	Global rather than cross-generational optimization	No fairness guarantee for legacy stations	Decrease in temporal predictability	(I) + (L) [13,14]
Support for low-throughput devices	Naturally well-suited	Secondary to high-throughput traffic	Mismatch with the IoT traffic profile	Goodput fluctuations despite low load	(E) + (I) [8,12]
Feedback transmission mechanisms	Classic ACK, backoff	Block ACK, uplink scheduling	Feedback delays for legacy stations	Increase in RTT and MQTT timeouts	
System scaling	Limited	Very good	Scalability not transferable to legacy stations	Quality degradation as density increases	
Temporal determinism	Limited	Better for HE stations	No cross-generation determinism	Jitter is the key problem	(E) + (L) [5,6]
Perceived quality of service	Acceptable in homogeneous networks	High in HE networks	QoS/QoE divergence between device classes	High PDR, but reduced QoE	(E) + (I)

## Data Availability

The original contributions presented in this study are included in the article. Further inquiries can be directed to the corresponding author.

## Supplementary Materials

The following supporting information can be downloaded at https://www.mdpi.com/article/10.3390/s26175352/s1, Figure S1: Application throughput over time (moving average over a 30 s window), Figure S2: Application throughput over time (moving average over a 120 s window).
